# Homologous Recombination in Protozoan Parasites and Recombinase Inhibitors

**DOI:** 10.3389/fmicb.2017.01716

**Published:** 2017-09-07

**Authors:** Andrew A. Kelso, Sarah M. Waldvogel, Adam J. Luthman, Michael G. Sehorn

**Affiliations:** ^1^Department of Genetics and Biochemistry, Clemson University, Clemson SC, United States; ^2^Eukaryotic Pathogens Innovation Center, Clemson University, Clemson SC, United States; ^3^Center for Optical Materials Science and Engineering Technologies, Clemson University, Clemson SC, United States; ^4^Clemson University School of Health Research, Clemson University, Clemson SC, United States

**Keywords:** homologous recombination, DNA repair, double-strand break repair, protozoan parasites, recombination inhibitors, RAD51, DMC1

## Abstract

Homologous recombination (HR) is a DNA double-strand break (DSB) repair pathway that utilizes a homologous template to fully repair the damaged DNA. HR is critical to maintain genome stability and to ensure genetic diversity during meiosis. A specialized class of enzymes known as recombinases facilitate the exchange of genetic information between sister chromatids or homologous chromosomes with the help of numerous protein accessory factors. The majority of the HR machinery is highly conserved among eukaryotes. In many protozoan parasites, HR is an essential DSB repair pathway that allows these organisms to adapt to environmental conditions and evade host immune systems through genetic recombination. Therefore, small molecule inhibitors, capable of disrupting HR in protozoan parasites, represent potential therapeutic options. A number of small molecule inhibitors were identified that disrupt the activities of the human recombinase RAD51. Recent studies have examined the effect of two of these molecules on the *Entamoeba* recombinases. Here, we discuss the current understandings of HR in the protozoan parasites *Trypanosoma*, *Leishmania*, *Plasmodium*, and *Entamoeba*, and we review the small molecule inhibitors known to disrupt human RAD51 activity.

## Introduction

DNA double-stranded breaks (DSBs) can occur due to exogenous or endogenous events. Exogenous sources of DSBs include reactive oxygen species generated from exposure to ionizing radiation and radiomimetic chemicals (Mehta and Haber, 2014), while endogenous sources of DSBs include erroneous DNA replication that can lead to the collapse of replication forks. Additionally, DSBs can be intentionally introduced into the genome through programmed events such as in meiosis, where the production of crossover products is essential. Whether they are produced intentionally or as a result of genotoxic events, unrepaired DSBs threaten the genome stability of an organism. Defects in DSB repair pathways have been associated with sterility, cancer, and chromosomal rearrangements (San Filippo et al., 2008; Moynahan and Jasin, 2010; Hunter, 2015).

**FIGURE 1:**
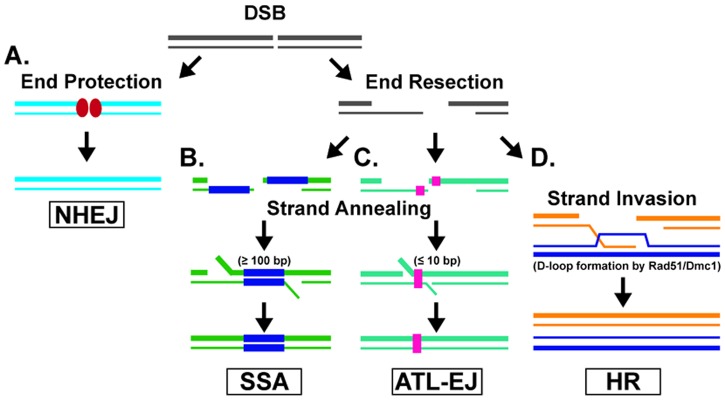
Double-strand Break Repair Pathways. **(A)** The non-homologous end-joining (NHEJ) pathway is facilitated when the ends of the DSB are protected from resection and then ligated to mend the DSB. **(B)** Single-strand annealing (SSA) is a DSB repair pathway that anneals long stretches of homologous sequences flanking the DSB site. **(C)** The alternative end-joining (ALT-EJ) pathway anneals microhomologous sequences next to the DSB site. **(D)** In the homologous recombination (HR) repair pathway, a recombinase (Rad51 and/or Dmc1 in most eukaryotes) utilizes a homologous template (i.e., sister chromatid) to faithfully repair the DSB.

Four major pathways function in DSB repair: non-homologous end joining (NHEJ), single-strand annealing (SSA), alternative end joining (ALT-EJ), and homologous recombination (HR) (**Figure 1**). NHEJ (also known as classical-NHEJ or canonical-NHEJ) is the major DSB repair pathway, and is mediated by many factors, which include Ku70, Ku80, XRCC4, and DNA ligase IV (Lieber, 2010). In this pathway, the ends of the DSB are protected to prevent nucleolytic end resection (Mimori and Hardin, 1986; Paillard and Strauss, 1991), followed by ligation to mend the DSB (**Figure 1A**). NHEJ is active during the entire cell cycle, and is the pathway responsible for V(D)J recombination in antibody variation (Soulas-Sprauel et al., 2007; Malu et al., 2012). NHEJ can result in full restoration of the DSB. However, if there is any enzymatic processing or degradation at the ends of the DSB, there will be a loss of genetic information once repaired (Betermier et al., 2014); thus, NHEJ is often associated with chromosomal alterations at the break site (Ghezraoui et al., 2014). Repair by SSA is initiated when a DSB occurs at a locus with extensive homology in the sequences flanking the break site (**Figure 1B**). Once homology is located (≥100 base pairs), the sequences are annealed by the DNA annealing factor RAD52 (Morales et al., 2015) and the non-homologous overlapping ends are endonucleolytically processed followed by DNA polymerase gap filling and ligation to restore the break (Bhargava et al., 2016). Similarly, ALT-EJ (also known as a microhomology mediated-EJ) DSB repair occurs through the recognition and annealing of short sequences called microhomology (≤10 base pairs) within the sequences flanking the DSB (Wang and Xu, 2017) (**Figure 1C**). The two main protein factors involved in mediating ALT-EJ are poly-ADP-ribose polymerase (PARP) and DNA polymerase theta (POLQ) (Bhargava et al., 2016). SSA and ALT-EJ are both active early in the S and G2 phases of the cell cycle. Both pathways result in the loss of DNA sequence between the annealed substrates (SSA results in large deletion events and ALT-EJ results in small deletions). As a result, both SSA and ALT-EJ are considered to be mutagenic DSB repair pathways (Bhargava et al., 2016). In contrast, HR relies on a homologous template to restore the damaged DNA in its entirety (**Figure 1D**). HR is active during the S and G2 phases of the cell cycle due to the presence of sister chromatids or homologous chromosomes (in meiosis) to act as the template. The HR pathway requires enzymatic resection of the ends of the DSB to produce 3′ single-stranded DNA (ssDNA) overhangs. These overhangs serve as the nucleation site for a recombinase, the central enzyme of HR. There are two conserved recombinases among eukaryotes, Rad51 (radiation-sensitive 51) and Dmc1 (disrupted meiotic cDNA 1) (Moore, 1978; Bishop et al., 1992). Rad51 is the lead recombinase during mitosis, and Dmc1 serves as the major recombinase during meiosis, in which Rad51 plays a supportive role (Bishop, 2012). These different processes result in the requirement for different accessory proteins to modulate the functions of each recombinase. Nonetheless, the mechanisms of action for both recombinases are similar. The active ATP-bound form of the recombinase forms a right-handed presynaptic, nucleoprotein filament on the ssDNA overhang. The presynaptic filament then conducts a homology search to identify a homologous sequence that will serve as a template for repair. Rad51 preferentially utilizes the sister chromatid as a template in order to prevent loss of heterozygosity, whereas Dmc1 prefers the homologous chromosome with the goal of generating crossover products. In either case, the presynaptic filament invades the duplex DNA and facilitates the base-pairing of the 3′ ssDNA with its complementary sequence. As a result, the homologous strand of the duplex DNA is displaced to form a displacement loop (D-loop) structure. The 3′ end of the invading strand primes DNA synthesis by a polymerase to replace DNA that was lost at the DSB site. As a result of strand invasion and DNA synthesis to repair the damaged DNA, two distinct DNA joint molecules can be formed. These DNA intermediates (the initial D-loop or Holliday junctions, which are associated with meiosis and form after D-loop extension, second end capture, ligation, and a second synthesis event) are resolved by one of two major pathways to ultimately yield non-crossover products or crossover products (Matos and West, 2014; Morrical, 2015).

The complex process of DSB repair by HR requires numerous accessory proteins to proceed with accuracy and efficiency (**Table 1**). RPA (replication protein A) is a heterotrimeric complex composed of three subunits (RPA1, RPA2, RPA3) that coat the ssDNA overhangs to protect them from nucleases and to prevent the formation of secondary structure (Binz et al., 2004). Although the DNA binding activity of RPA is necessary for HR, this function also represents a barrier to the recombinase loading. Various recombination mediators help to overcome this inhibition by displacing RPA and loading the recombinase onto the ssDNA, including *Saccharomyces cerevisiae* Rad52 (radiation-sensitive 52) and human BRCA2 (breast cancer susceptibility gene 2) (Sung, 1997; Jensen et al., 2010; Liu et al., 2010; Thorslund et al., 2010). Recently, DSS1 (deleted in split hand/split foot syndrome) was shown to aid BRCA2 in loading RAD51 onto RPA-coated ssDNA (Yang et al., 2002; Zhao et al., 2015). DSS1 functions as a DNA mimic that competes with authentic DNA to displace RPA, allowing BRCA2 to load RAD51 onto ssDNA (Zhao et al., 2014). Another factor, Hop2-Mnd1 (homologous-pairing protein 2—meiotic nuclear division protein 1), is a meiosis-specific heterodimeric protein complex that promotes recombinase-meditated D-loop formation by stabilizing the presynaptic filament and bringing the duplex DNA into close proximity with the presynaptic filament for more efficient homologous DNA pairing (Chi et al., 2007; Pezza et al., 2007). Other accessory proteins, such as RAD54 (radiation-sensitive 54), promote the search for homology by the recombinase and help to dissociate the recombinase from the DNA after strand exchange has occurred (Petukhova et al., 1998; Mason et al., 2015). The high degree of conservation of the HR pathway across divergent eukaryotic species emphasizes its indispensable nature. The clinical relevance of HR in non-communicable human disease, such as cancer, makes it a potential therapeutic target. Furthermore, since HR plays a role in virulence of eukaryotic pathogens (see below), the components of this DNA repair system may also serve as a target for the development of new drugs to fight infectious disease.

**Table 1:** HR DSB repair proteins, species, functions, and UniProtKB identifiers of human, yeast, and putative protozoan parasite homologs.

Function	*Homo sapiens*	*Saccharomyces cerevisiae*	*Entamoeba histolytica*	*Trypanosoma brucei*	*Plasmodium falciparum*	*Leishmania major*
DSB End Resection	MRE11	Mre11	C4LVX7	Q8T8P1–?	A0A1C3KMQ6	E9ADG7
	RAD50	Rad50	N9V1K1	Q384J8	C6KSQ6	Q4Q8L7
	NSB1	Xrs2	?	?	?	?
Single-strand DNA Binding	RPA1-RPA2-RPA3	Rfa1-Rfa2-Rfa3	?-?-?	?-?-?	A0A1C3KN47/A0A1C3KQ72	?-?-?
Recombinase (Meiosis-specific)	RAD51	Rad51	Q86C17^∗^	Q384K0^∗^	Q8IIS8	O61127
	DMC1	Dmc1	C4LTR6^∗^	Q38E34^∗^	A0A1C3KPB3	O61128
Accessory Proteins	RAD54	Rad54	C4LVM6^∗^	Q385M5	Q8IAN4	Q4QH75
	RAD54B	Rdh54	N9TAM9	?	?	?
	RAD55	Rad55	?	?	?	?
	RAD57	Rad57	?	?	?	?
	–	RAD59	?	?	?	?
	HOP2	Hop2	?	?	?	?
	MND1	Mnd1	M3TLC0	?	C6S3J7	Q4QAN2
Single-Strand Annealing	RAD52	Rad52	C4M197	?	?	?
Mediator	BRCA2	Rad52	N9TLS7	Q4GZF5	?	Q4QD38

^∗^*Indicates characterized enzymes parasites*.

*?Indicates no known homolog*.

## Homologous Recombination in Protozoan Parasites

Homologous recombination is essential in many protozoan parasites. These parasites utilize HR to adapt to diverse environmental conditions, evade host immune systems, and respond to DSBs (Deitsch et al., 1997; Bhattacharyya et al., 2004). Many of the core eukaryotic enzymes involved in HR are conserved among protozoan parasites (**Table 1**); however, the mechanisms of HR in many of these pathogens are largely unknown. Over the past few years, our understanding of HR in these organisms has increased from studies involving *Trypanosoma*, *Leishmania*, *Plasmodium*, and *Entamoeba.*

**FIGURE 2:**
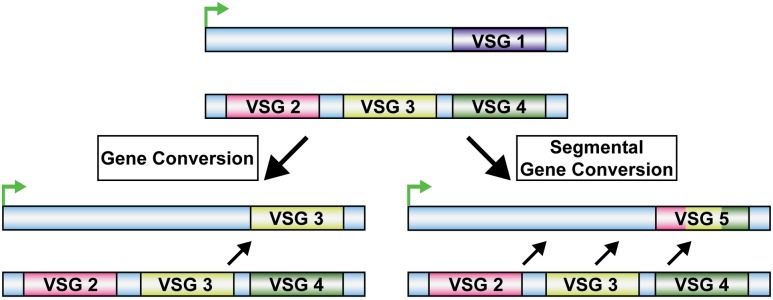
*Trypanosoma brucei* Homologous Recombination VSG Switching. VSG Switching by homologous recombination in *T. brucei* occurs through gene conversion. This can occur by exchanging a VSG gene into a transcriptionally active site or through multiple VSG gene segment conversions into the transcriptionally active site.

Homologous recombination plays a significant role in the survival of *T. brucei*, the protozoan parasite responsible for African trypanosomiasis (sleeping sickness). *T. brucei* generates antigenic variation through DNA recombination to evade the host immune system (McCulloch and Barry, 1999). In *T. brucei*, antigenic variation consists of repeatedly changing the expression of the Variant Surface Glycoprotein (VSG) genes that encode an outer protective coat of the parasite (Cross, 1975). *T. brucei* expresses more than 1000 VSG genes and pseudogenes one at a time (Borst, 2002; Marcello and Barry, 2007; McCulloch et al., 2015). One of the ways in which *T. brucei* can switch the expression of the VSG gene is through gene conversion of entire VSG genes or partial VSG pseudogenes into a transcriptionally active site using HR (**Figure 2**) (Borst, 2002; Marcello and Barry, 2007; Vink et al., 2012). It was shown that Rad51 was a major factor in this process, as null mutations of Rad51 in *T. brucei* (*Tb*Rad51) led to a reduction in the VSG switching frequency (McCulloch and Barry, 1999). Moreover, mutations in genes involved in other DSB repair pathways (i.e., NHEJ) induced no change in VSG switching frequency (Conway et al., 2002a), indicating that VSG switching is likely dependent on *Tb*Rad51. However, the frequency of VSG switching was reduced rather than eliminated in *Tb*Rad51 mutants, suggesting that RAD51-mediated HR is not the only pathway for VSG switching (McCulloch and Barry, 1999; Conway et al., 2002a,b). Indeed, ALT-EJ also exists as an alternative pathway to HR in this pathogen (Conway et al., 2002b; Barnes and McCulloch, 2007; Glover et al., 2008, 2011). Furthermore, *Tb*Rad51 was reported to be important in response to DNA damage. When DSBs were induced in *T. brucei*, *Tb*Rad51 was shown to localize to sub-nuclear foci (Glover and Horn, 2012), and HR served as the predominant DSB repair pathway (Barnes and McCulloch, 2007; Glover et al., 2008).

*Trypanosoma cruzi* is a protozoan parasite that causes Chagas disease, or American trypanosomiasis. *T. cruzi* Rad51 (*Tc*Rad51) has a significant role in the response to and repair of DNA damage caused by ionizing radiation, as shown by *in vivo* analyses (Regis-da-Silva et al., 2006). Interestingly, the genomes of *T. brucei* and *T. cruzi* contain Dmc1 genes with 65% and 70% identity to human DMC1, respectively (Proudfoot and McCulloch, 2006; Kelso et al., 2017). However, heterozygous and homozygous knockout *DMC1* mutants (*DMC1*^+/-^ and *dmc1*^-/-^, respectively) in *T. brucei* were not sensitive to DNA damage, failed to impact VSG switching frequency, and had unaltered recombination efficiency, suggesting that *Tb*Dmc1 played no role in these processes (Proudfoot and McCulloch, 2006). These results were in direct contrast to the studies of *T. brucei rad51*^-/-^ mutants (McCulloch and Barry, 1999; Conway et al., 2002b). Currently, there is no evidence to suggest that *Tb*Rad51 compensates for the loss of *Tb*Dmc1, or vice versa. Future work will be necessary to reveal the biochemical underpinnings of Rad51 and Dmc1 recombinases from *T. brucei* and *T. cruzi*.

Leishmaniasis is a diverse disease characterized by three main forms: fever/anemia/weight loss, skin lesions, or mucous membrane destruction. Leishmaniasis is caused by the protozoan parasite *Leishmania*. *Leishmania* utilize HR to induce gene rearrangement events in response to oxidative stress, while HR-mediated DNA amplification events serve as a mechanism for drug resistance (Beverley, 1991; Ouellette and Papadopoulou, 1993; Nathan and Shiloh, 2000; Ubeda et al., 2008, 2014; Monte-Neto et al., 2015). Although HR is exploited *in vitro* to create null mutants of *L. major* for study (Cruz et al., 1991), the mechanisms of HR in *L. major* are largely unknown. There are a few studies demonstrating the existence of functional Rad51 in *Leishmania.* Namely, *in vivo* studies of *L. major* showed that the typically low levels of Rad51 (*Lm*Rad51) expression increased in response to the DNA damaging agent phleomycin (McKean et al., 2001). Similarly, *L. infantum* Rad51 (*Li*RAD51) was highly expressed in response to phleomycin exposure, while the expression levels of the *L. infantum* ortholog of BRCA2 (*Li*BRCA2) were unchanged, similar to what has been reported in human cells (Lu et al., 2005). *Li*RAD51 and *Li*BRCA2 both localize to punctate foci in the nucleus; however, *Li*RAD51 fails to localize to the nucleus in *Li*BRCA2-deficient cells (Genois et al., 2012). Biochemically, *Lm*Rad51 ATP hydrolysis activity was stimulated by the presence of both ssDNA and dsDNA (McKean et al., 2001). Another biochemical study showed that *Li*RAD51 binds DNA—with an apparent higher affinity for ssDNA over dsDNA (Genois et al., 2012), and *Li*RAD51 can facilitate homologous DNA pairing (Genois et al., 2012). Furthermore, *Li*BRCA2 was reported to load *Li*RAD51 onto RPA coated ssDNA (Genois et al., 2012), similar to its human homolog (Jensen et al., 2010; Liu et al., 2010; Thorslund et al., 2010).

*P. falciparum* causes malaria in humans. The genome of *P. falciparum* contains many of the HR proteins (**Table 1**) (Gardner et al., 2002; Kirkman et al., 2014). *In vivo* studies using an exogenous HR reporter substrate with an inducible DSB site found that HR was the preferred DSB repair pathway when a homologous template was available, and during the more common haploid state, ALT-EJ was preferred (Kirkman et al., 2014). Upon exposure to the DNA damaging agent, methyl methanesulfonate (MMS), *P. falciparum* Rad51 (*Pf*Rad51) expression increased (Bhattacharyya and Kumar, 2003). Biochemical analysis of *Pf*Rad51 showed that it hydrolyzed ATP and facilitated efficient DNA strand exchange (Bhattacharyya et al., 2005). *Pf*Rad51 required ATP binding but not hydrolysis to catalyze DNA strand exchange *in vitro* (Bhattacharyya et al., 2005), similar to human RAD51. In support of this notion, mutational analysis of the ATP binding motif of *Pf*Rad51 showed that *Pf*Rad51K143R significantly impacted the *in vivo* function of *Pf*Rad51 (Roy et al., 2014). Furthermore, *P. berghei* (responsible for malaria in rodents) and *S. cerevisiae* expressing the *Pf*Rad51-K143R variant were found to be hypersensitive to MMS treatment (Roy et al., 2014).

In addition to the studies on *Pf*Rad51, homologs of other HR proteins were shown to have similar functions and/or responses to DNA DSBs in *Plasmodium* as reported for their human counterparts. For example, Rad54 is a Rad51 stimulating protein, and *Pf*Rad54 similarly stimulated the homologous DNA pairing activity of *Pf*Rad51 (Gopalakrishnan and Kumar, 2013). BLM (Bloom syndrome protein) is a DNA helicase involved in DSB end-resection, and *Pf*Blm possessed helicase activity (Rahman et al., 2016). The protein complex Mre11 (meiotic recombination 11), Rad50 (radiation-sensitive 50), and NBS1 (involved in Nijmegen breakage syndrome) recognizes and end-resects DNA DSBs, and *Pf*Mre11 demonstrated nuclease activity and interacted with *Pf*Rad50 (Badugu et al., 2015). Lastly, diploid zygotes of *Plasmodium* undergo meiosis in the insect vector to produce haploid cells, a state in which Dmc1 becomes relevant. One study in *P. berghei* null for *dmc1* demonstrated that *Pb*Dmc1 was essential for proper oocyst development (Mlambo et al., 2012). *Pb*Dmc1 null cells were also shown to be highly sensitive to a DNA damaging agent (Mlambo et al., 2012).

**FIGURE 3:**
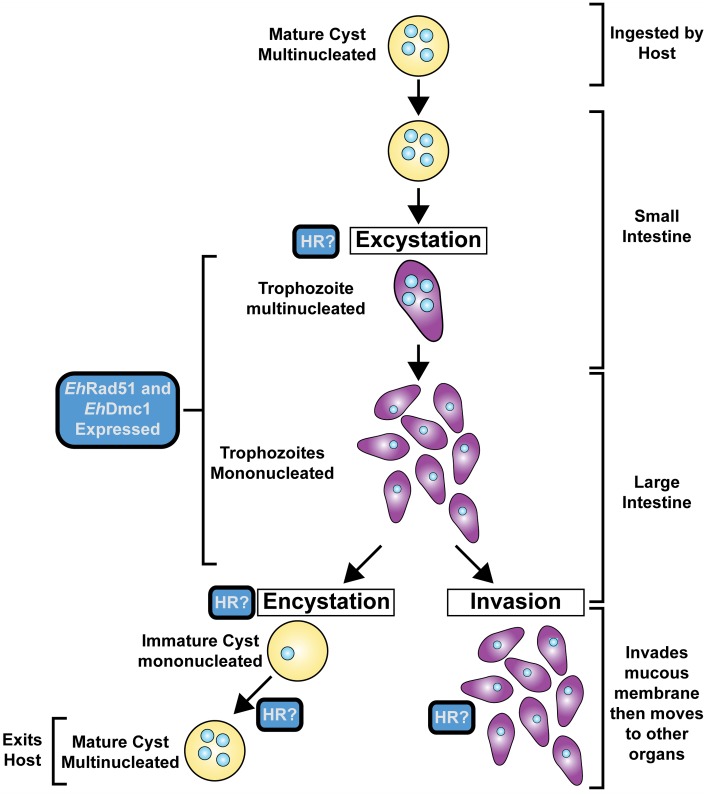
Life Cycle of *Entamoeba histolytica*. The human host ingests the mature cyst. The cyst moves to the small intestine where excystation occurs, resulting in the release of multiple trophozoites that migrate to the large intestine. Trophozoites either invade the mucous membrane of the host or encyst and exit the host for future infections.

*Entamoeba histolytica* is the parasite responsible for amoebic dysentery and amoebic liver abscess. The life cycle of *E. histolytica* consists of a tetra-nucleated cyst that is transmitted through water or food sources by fecal contamination (**Figure 3**). When ingested by the host, the mature, tetra-nucleated cyst can undergo excystation in the small intestine resulting in the release of eight trophozoites that can colonize the large intestine. Trophozoites multiply by binary fission and produce multinucleated cysts through the process of encystation (Dobell, 1928; Cleveland and Sanders, 1930; Ratcliffe and Geiman, 1934; Koushik et al., 2014). There is ample support for HR in *E. histolytica.* For example, the genome of *E. histolytica* contains the majority of the HR core genes (**Table 1**) (Bhattacharya et al., 2000). Also, genome duplication events, unscheduled gene amplification events, and genetic rearrangements are reported in *E. histolytica* and thought to be mediated by HR (Orozco et al., 1988; Baez-Camargo et al., 1996; Bhattacharya et al., 2000; Mukherjee et al., 2008). The first study on HR in *E. histolytica* monitored the response of *E. histolytica* to UV-C radiation, which is known to cause DNA damage (Lopez-Casamichana et al., 2008). Many hallmarks of HR were observed upon DNA damage induction in *E. histolytica*: the histone H2AX was phosphorylated (indicating DNA damage), cell survivability was not affected (indicating DSB repair occurred), the core HR genes were differentially expressed in response to irradiation, *Eh*Rad51 expression (mRNA and protein) peaked rapidly in response to DNA damage, and *Eh*Rad51 formed nuclear foci in response to DNA damage (Lopez-Casamichana et al., 2008; Charcas-Lopez Mdel et al., 2014). During growth stresses (heat shock, oxygen stress, serum starvation) and in response to DNA damage (UV irradiation), inverted repeat sequences located either on a plasmid or in the *E. histolytica* genome underwent recombination (Singh et al., 2013). This represents the first direct evidence for HR in *E. histolytica*. Biochemically, Lopez-Casamichana et al. (2008) showed that partially purified *Eh*Rad51 bound DNA and facilitated homologous DNA pairing (Lopez-Casamichana et al., 2008). The findings were recently extended by a report that demonstrated *Eh*Rad51 was not only capable of binding DNA and forming D-loops, but that *Eh*Rad51 hydrolyzed ATP, formed presynaptic filaments, and catalyzed DNA strand exchange over thousands of base pairs (Kelso et al., 2016). Although *E. histolytica* reproduce asexually through binary fission, the occurrence of meiosis in *E. histolytica* was first proposed to occur during encystation based on increased expression of meiosis-specific genes (Ehrenkaufer et al., 2013). Additional support for this notion came from monitoring the expression of meiosis-specific genes in the reptilian amoebozoa parasite, *E. invadens* during encystation (Singh et al., 2013). More recently, biochemical analysis of *Eh*Dmc1 demonstrated the enzyme hydrolyzed ATP in the presence of DNA, bound DNA, formed nucleoprotein filaments, and catalyzed homologous DNA pairing and DNA strand exchange (Kelso et al., 2015). Taken together, the evidence suggests that mitotic and meiotic HR occur and are likely important for encystation in *Entamoeba* (**Figure 3**).

Currently, the roles of other DSB repair pathways in protozoan parasites are largely uncharacterized. As mentioned above, ALT-EJ has been reported for some parasites, but the mechanisms remain elusive (Conway et al., 2002b; Barnes and McCulloch, 2007; Glover et al., 2008, 2011; Kirkman et al., 2014). As for SSA, although many genomes of protozoan parasites contain the *RAD52* gene, *T. brucei* is the only pathogen to demonstrate a potential for SSA (Glover and Horn, 2009). Lastly, the genomes of some parasites, like *T. brucei* and *E. histolytica*, contain factors necessary for NHEJ (e.g., Ku); however, many of the key components of this pathway are missing such as DNA ligase IV and XRCC4 homologs (Burton et al., 2007). In fact, *T. brucei* Ku was shown to have a role in telomere length maintenance, but DSB repair occurred independent of Ku (Conway et al., 2002a).

## Targeting Homologous Recombination with Small Molecule Inhibitors

Studies of small molecule inhibitors targeting the proteins involved in HR have increased in recent years (Bryant et al., 2005; Ishida et al., 2009; Huang et al., 2011, 2012; Takaku et al., 2011; Budke et al., 2012, 2013; Huang and Mazin, 2014; Normand et al., 2014). Many current anticancer therapies, such as irradiation and chemotherapy, are designed to induce DNA damage and provoke an apoptotic response. By targeting major HR enzymes like RAD51, RAD54, and BRCA2, the susceptibility of cancer cells could increase when combined with other DNA-damaging therapies. An analogous argument could be made for the targeting of enzymes involved in HR in protozoan parasites, which rely on HR for diverse environmental adaptations, host immune evasion, and drug resistances (Deitsch et al., 1997; Bhattacharyya et al., 2004).

**FIGURE 4:**
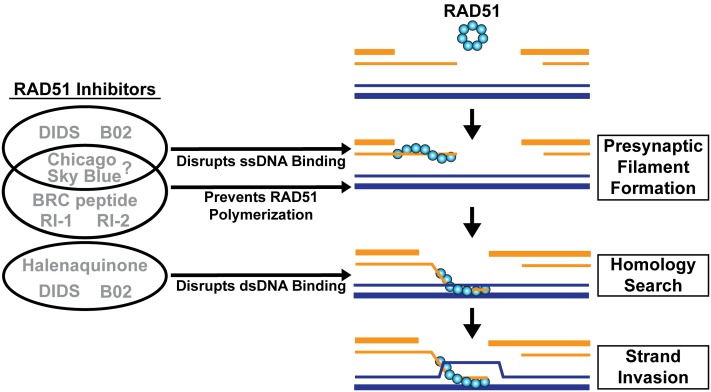
Inhibitors of human RAD51. RAD51 inhibitors can prevent RAD51 from self-associating or from binding ssDNA and/or dsDNA. DIDS and B02 disrupt ssDNA and dsDNA binding. Chicago Sky Blue prevents filament formation but it is unknown if this is through inhibition of ssDNA binding or RAD51 polymerization. RI-1 and RI-2 block RAD51 polymerization binding sites, and the BRC peptide binds filament-dissociated RAD51 monomers, leading to inhibition. Halenaquinone disrupts the dsDNA binding of RAD51.

RAD51 serves as a reasonable drug target for protozoan parasites because it is important for the repair of damaged DNA by HR. However, for RAD51 to be an effective target for small molecule inhibitors in these pathogens, it is essential to develop inhibitors that are species-specific, since RAD51 is also ubiquitously expressed in the human host. Currently, there is limited structural information about the recombinases, which hinders the potential for structure-based drug design. Due to the availability of purified recombinases, high throughput screens could provide a powerful platform for identifying potential compounds that demonstrate efficient inhibition of parasitic recombinases but not human recombinases. Additionally, there are many well-characterized biochemical assays that could act as secondary analyses to aid in the understanding of mechanisms of inhibition. On a related note, recent studies have utilized small molecules, previously demonstrated to inhibit human RAD51, to target the activities of the *E. histolytica* recombinases (Kelso et al., 2015, 2016). The small molecule 4,4′-diisothiocyanostilbene-2,2′-disulfonic acid (DIDS) was found to directly interact with human RAD51 and interfere with its ability to bind ssDNA and dsDNA (**Figure 4**) (Ishida et al., 2009). By interfering with the ability of RAD51 to bind DNA, DIDS decreases the presynaptic filament formation of RAD51, which is essential for DNA strand exchange, D-loop formation, and ATP hydrolysis (Ishida et al., 2009). This small molecule was also shown to disrupt the recombinase activities of both *Eh*Dmc1 and *Eh*Rad51 *in vitro* (Kelso et al., 2015, 2016). Notably, encystation was significantly hindered in *E. invadens* when DIDS was present (Kelso et al., 2016). Despite its efficacy in these pathogens, DIDS exhibits high toxicity for human cells, complicating its use as a potential anti-pathogen treatment (Ishida et al., 2009). Interestingly, in the same study by Kelso et al. (2016), another human RAD51 small molecule inhibitor, B02, was tested. B02 was reported to disrupt RAD51 binding to ssDNA during presynaptic filament formation. In addition, B02 disrupts the subsequent binding of dsDNA to the RAD51-ssDNA complex (**Figure 4**) (Huang et al., 2011). As a result, B02 inhibits human RAD51-mediated DNA strand exchange and D-loop formation activities (Huang et al., 2011). *In vivo* studies revealed that B02 also prevents RAD51 foci formation at DSBs and effectively sensitizes human cells to DNA damage by the interstrand crosslinking agents cisplatin and mitomycin-C and to PARP1 inhibitors, which target the PARP1 system responsible for repair of ssDNA breaks (Huang et al., 2011, 2012; Huang and Mazin, 2014). When tested with *Eh*Dmc1 and *Eh*Rad51, B02 failed to disrupt their DNA strand exchange activity at concentrations that inhibited human RAD51 (Kelso et al., 2016). This was the first study to demonstrate species selectivity by small molecule inhibitors of human and protozoan recombinases. Importantly, these findings provide evidence that inhibitors could potentially be tailored to target specific recombinases.

There are additional small molecule inhibitors reported to disrupt human RAD51 activities that have not been characterized for their efficacy against protozoan recombinases. Halenaquinone disrupts the ability of RAD51 to bind dsDNA regardless of the presence of ssDNA (Takaku et al., 2011). This suggests that the compound binds to the dsDNA binding site, severely compromising the ability of RAD51 to catalyze homologous DNA pairing (**Figure 4**). Thus, *in vivo* studies demonstrated that halenaquinone suppressed the formation and retention of RAD51 foci at DSB sites (Takaku et al., 2011). Although halenaquinone interacts with RAD51 in a manner similar to DIDS, it does not exhibit the same level of toxicity for human cells (Takaku et al., 2011).

RI-1 is a compound that was shown to inhibit human RAD51 presynaptic filament formation by irreversibly binding to cysteine-319 at the oligomerization interface (**Figure 4**) (Budke et al., 2012). By inhibiting monomer-monomer interaction, RI-1 prevents polymerization of the RAD51 filament onto ssDNA and consequently inhibits the assembly of RAD51 foci *in vivo* (Budke et al., 2012). Additionally, RI-1 was shown to sensitize cancer cells to mitomycin-C, a compound that induces DNA damage via cross-linking. RI-1 reacts with thiol groups (hence conjugation to cysteine-319) and therefore has the potential for off-target effects. Thus, RI-2, a derivative of RI-1, was developed to inhibit RAD51 via the same mechanism but in a reversible capacity in order to reduce the likelihood of serious off-target binding effects (**Figure 4**) (Budke et al., 2013). RI-2 was also shown to have a longer half-life than RI-1 and is a functional inhibitor of RAD51 in human cells. However, due to the nature of its reversible binding to the cysteine-319 of RAD51, RI-2 requires higher concentrations to achieve the same efficacy as the irreversibly bound RI-1 (Budke et al., 2013).

Chicago Sky Blue (CSB) is a potent inhibitor of human RAD51 activity, with sufficient inhibition observed in the 400 nM range (Normand et al., 2014). CSB inhibits D-loop formation and DNA strand exchange activity of RAD51 by preventing filament assembly of RAD51 onto ssDNA, effectively inhibiting its HR activities (Normand et al., 2014). However, it is unclear if the suppression of RAD51-ssDNA binding occurs by interfering with the ability of RAD51 to bind ssDNA (as seen with DIDS) or by disruption of protein polymerization onto ssDNA via binding at the protein–protein interface (as seen with RI-1 and RI-2) (**Figure 4**). Importantly, CSB exhibits the lowest IC_50_ (400 nM) of the RAD51 inhibitory compounds, making it a potent RAD51 inhibitor with significant clinical potential (Normand et al., 2014).

Lastly, a short peptide of the BRC4 repeat from human BRCA2 was shown to dissociate the RAD51 filament by binding to the filament-dissociated monomers of RAD51 thereby rendering RAD51 inactive through sequestration (**Figure 4**) (Nomme et al., 2008).

Given that RAD51 is highly conserved among eukaryotes and is ubiquitously expressed, other HR proteins might serve as alternative therapeutic targets. Currently, HR in protozoan parasites is an emerging field, and therefore, the enzymes involved are not well understood. As this field develops, new HR targets that are essential and specific to parasites may emerge. Consequently, identifying if these exist in protozoan parasites would be valuable for potential pathogenic targets.

## Conclusion and Perspectives

Homologous recombination is an essential DNA repair process. Dysfunction of this pathway can challenge the viability and fecundity of an organism. The majority of the HR repair machinery is highly conserved among eukaryotes, including RAD51. RAD51 is responsible for DNA strand exchange and homology search via the formation of a nucleoprotein filament on ssDNA overhangs after end resection of a DSB. As a result of its central role in HR, there is merit for considering RAD51 as potential therapeutic targets in human pathogens. Presently, there is no evidence to suggest that protozoa can use the repair machinery of their host; thus, targeting protozoan RAD51 remains feasible. Interestingly, one study demonstrated the ability of a mammalian accessory protein, murine Hop2-Mnd1, to stimulate the recombinase activities of *Eh*Rad51, so it is reasonable to consider the possibility. Further studies will be necessary to examine this hypothesis. Many of the RAD51 inhibitors reviewed here (DIDS, Halenaquinone, B02, CBS, BRC peptide) impede the interaction between RAD51 and DNA. As this function is essential for RAD51 filament formation and localization to DSB sites, it is an effective target to achieve inhibition of RAD51 activity in HR. Another class of RAD51 inhibitors (including RI-1 and RI-2) disrupt presynaptic filament formation by physically preventing RAD51 polymerization onto ssDNA. Again, inhibition of RAD51 at this early HR stage abolishes repair of DSBs, and therefore can lead to cell death. The ability to disrupt RAD51 in parasites and not inhibit human RAD51 will be critical for the development of new species-specific small molecule inhibitors. Analysis of the targeted impact of some of these molecules on *Eh*Rad51 versus human RAD51 suggests that there is some degree of variance between the homologs that allows molecule specificity (note the specificity of B02 for human RAD51). Since protozoan parasites rely on HR for host immune evasion and drug resistance, targeting the major enzymes involved in the HR pathway could lead to new therapeutic intervention strategies. For example, under stressful environmental conditions, *E. histolytica* can undergo encystation for survival, which involves multiple rounds of DNA replication that produces a polyploid cyst. It has been suggested that HR plays a substantial role in encystation in *E. histolytica*, which has been modeled in the related reptilian parasite, *E. invadens*. Thus, inhibition of *Entamoeba* HR via *Eh*Rad51 small molecule inhibitors could limit *Entamoeba* pathogenicity by obstructing the encystation pathways. Future studies are necessary to determine the efficacy of these human recombinase inhibitors on the survivability of protozoan parasites. Additionally, small molecule inhibitor studies on human RAD51 could provide a platform for future compound design against the RAD51 recombinase from other organisms.

## Author Contributions

AK and MS conceived and designed the study. AK, SW, AL, and MS wrote and edited the manuscript. All authors approved the content of the manuscript for accuracy.

## Conflict of Interest Statement

The authors declare that the research was conducted in the absence of any commercial or financial relationships that could be construed as a potential conflict of interest.
